# Diaqua­tetra­bromidotin(IV) trihydrate

**DOI:** 10.1107/S1600536812036434

**Published:** 2012-08-25

**Authors:** Fei Ye, Hans Reuter

**Affiliations:** a Institut für Chemie, Anorganische Chemie II, Universität Osnabrück, Barbarastrasse 7, 49069 Osnabrück, Germany

## Abstract

The title compound, [SnBr_4_(H_2_O)_2_]·3H_2_O, forms large colourless crystals in originally sealed samples of tin tetra­bromide. It constitutes the first structurally characterized hydrate of SnBr_4_ and is isostructural with the corresponding ­hydrate of SnCl_4_. It is composed of Sn^IV^ atoms octa­hedrally coordinated by four Br atoms and two *cis*-related water mol­ecules. The octa­hedra exhibit site symmetry 2. They are arranged into columns along [001] *via* medium–strong O—H⋯O hydrogen bonds involving the two lattice water mol­ecules (one situated on a twofold rotation axis) while the chains are inter­connected *via* longer O—H⋯Br hydrogen bonds, forming a three-dimensional network.

## Related literature

For background information on hydrates of SnBr_4_, see: Pfeiffer (1914 ▶); Pickering (1895 ▶); Rayman & Preis (1884 ▶). For the isostructural compound [SnCl_4_(H_2_O)_2_]·3H_2_O, see: Shihada *et al.* (2004 ▶). For structural information on other hydrates of SnCl_4_, see: Semenov *et al.* (2005 ▶); Genge *et al.* (2004 ▶).

## Experimental

### 

#### Crystal data

[SnBr_4_(H_2_O)_2_]·3H_2_O
*M* *_r_* = 528.41
Monoclinic, 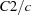

*a* = 12.6484 (5) Å
*b* = 10.1647 (4) Å
*c* = 8.8683 (4) Å
β = 104.462 (1)°
*V* = 1104.04 (8) Å^3^
*Z* = 4
Mo *K*α radiation
μ = 16.77 mm^−1^
*T* = 100 K
0.18 × 0.15 × 0.12 mm

#### Data collection

Bruker APEXII CCD diffractometer
Absorption correction: multi-scan (*SADABS*; Bruker, 2009 ▶) *T*_min_ = 0.148, *T*_max_ = 0.234
16999 measured reflections
1618 independent reflections
1523 reflections with *I* > 2σ(*I*)
*R*_int_ = 0.033

#### Refinement

*R*[*F* ^2^ > 2σ(*F* ^2^)] = 0.013
*wR*(*F* ^2^) = 0.028
*S* = 1.10
1618 reflections
51 parameters
H-atom parameters constrained
Δρ_max_ = 0.46 e Å^−3^
Δρ_min_ = −0.41 e Å^−3^

### 

Data collection: *APEX2* (Bruker, 2009 ▶); cell refinement: *SAINT* (Bruker, 2009 ▶); data reduction: *SAINT*; program(s) used to solve structure: *SHELXS97* (Sheldrick, 2008 ▶); program(s) used to refine structure: *SHELXL97* (Sheldrick, 2008 ▶); molecular graphics: *DIAMOND* (Brandenburg, 2006 ▶) and *Mercury* (Macrae *et al.*, 2008 ▶); software used to prepare material for publication: *SHELXTL* (Sheldrick, 2008 ▶).

## Supplementary Material

Crystal structure: contains datablock(s) I, global. DOI: 10.1107/S1600536812036434/fj2592sup1.cif

Structure factors: contains datablock(s) I. DOI: 10.1107/S1600536812036434/fj2592Isup2.hkl

Additional supplementary materials:  crystallographic information; 3D view; checkCIF report

## Figures and Tables

**Table 1:** Selected bond lengths (Å)

Sn1—O1	2.1378 (12)
Sn1—Br2	2.5420 (2)
Sn1—Br1	2.5439 (2)

**Table 2:** Hydrogen-bond geometry (Å, °)

*D*—H⋯*A*	*D*—H	H⋯*A*	*D*⋯*A*	*D*—H⋯*A*
O3—H3⋯Br2^i^	0.96	2.67	3.5239 (16)	148
O2—H22⋯Br2^ii^	0.96	2.92	3.6365 (14)	133
O2—H22⋯Br1^ii^	0.96	2.58	3.4004 (13)	144
O2—H21⋯O3^iii^	0.96	1.82	2.7595 (19)	167
O1—H12⋯O2^iv^	0.96	1.82	2.7182 (18)	155
O1—H11⋯O2	0.96	1.80	2.7318 (18)	164

Symmetry codes: (i) 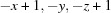; (ii) 
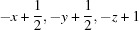
; (iii) 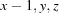; (iv) 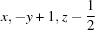.

**Fig. 1.**
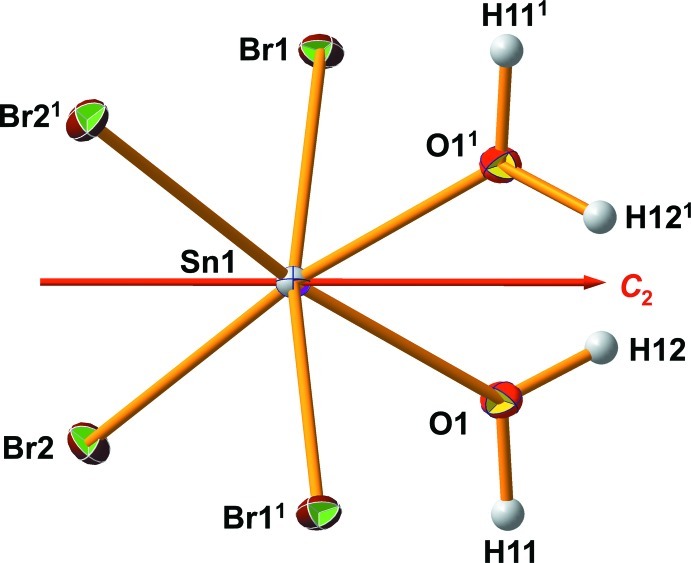
Ball-and-stick model of the {SnBr4(H2O)2} octahedron showing the atom labeling scheme used and the orientation of the twofold (C2) rotation axes. Displacement ellipsoids are drawn at the 50% probability level. Symmetry code: 1) -x, y, 1/2 - z.

**Fig. 2.**
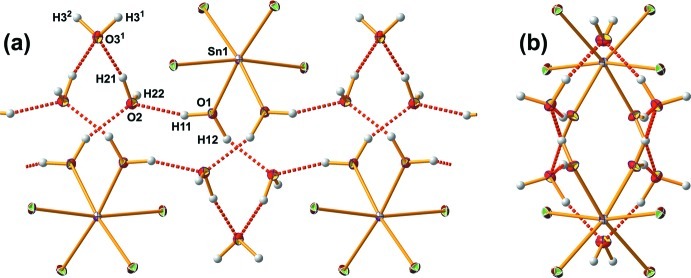
Schematic representation of the O—H···O hydrogen bonds (red broken lines) beetween the {SnBr4(H2O)2} octahedrons and the uncoordinated water molecules. (a) View perpendicular to, (b) into their tubelike arrangement. Symmetry codes: 1) -1 + x, y, z; 2) 1 - x, y, 3/2 - z.

**Fig. 3.**
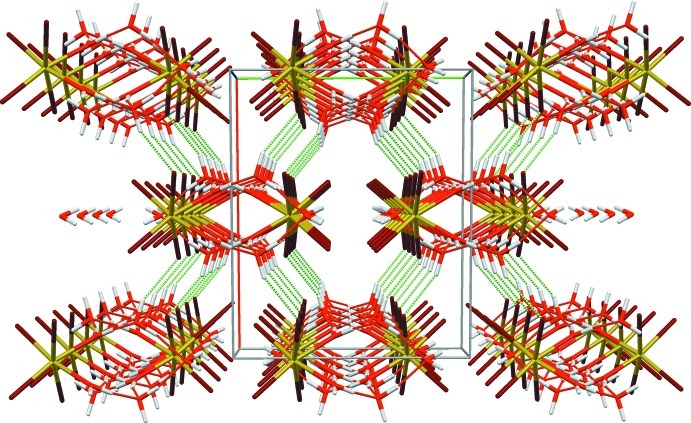
Tube packing as capped sticks model with the shortest O—H···Br hydrogen bridges (green broken lines) interconnecting neighboring tubes in the third dimension.

## supplementary crystallographic information

### Comment

In contrast to tin(IV) chloride, SnCl_4_, from which the crystal structures of hydrates, SnCl_4_^.^*n*H_2_O, with *n* = 2 (Semenov *et al.*, 2005), *n* = 3 (Genge *et al.*, 2004; Semenov *et al.*, 2005), *n* = 4 (Genge *et al.*, 2004; Shihada *et al.*, 2004), and *n* = 5 (Shihada *et al.*, 2004) are known in detail, no structural data are available concerning the hydrates of tin(IV) bromide, SnBr_4_^.^*n*H_2_O, although there are some hints in the older literature that a tetrahydrate, *n* = 4, (Rayman & Preis, 1884; Pfeiffer, 1914) and octahydrate, *n* = 8, (Pickering, 1895) exist. By chance, we found near the neck of some several years old, originally sealed bottles of tin(IV) bromide some large, colorless, transparent single-crystal in shape totally different from those of SnBr_4_ at the bottom of the flask. From single-crystal X-ray diffraction experiments we were able to confirm their composition to correspond to a so far unknown pentahydrate of tin tetrabromide, SnBr_4_^.^5H_2_O, 1.

The asymmetric unit of the title compound consists of half a {SnBr_4_(H_2_O)_2_} octahedron with tin at a twofold rotation axes, and one and a half water molecule in general position, respectively, on a twofold rotation axes, too. The {SnBr_4_(H_2_O)_2_} octahedron (Fig. 1) belongs to point group *C_2_* with the two coordinated water molecules in a *cis* position, a structural feature that is common to all the hydrates of SnCl_4_, mentioned above. Since the title compound is isostructural to the corresponding pentahydrate of SnCl_4_, 2, the influence of the larger bromine atom on bond angles and bond lengths can be studied in more detail. Both different tin-bromine distances are very similar and differ by only 0.0002 (2) Å, the mean value being 2.5435 (2) Å. The corresponding tin-chlorine distances were found to be 2.3824 (6) and 2.3826 (7) Å. The tin-oxygen distance of 2.135 (1) Å in 1 is somewhat longer than in 2 [2.115 (2) Å] but in the range [2.106 (5), tetrahydrate, T = 130 K, - 2.168 (2), dihydrate, T = 150 K] found in the other hydrates of SnCl_4_. Influence of the larger bromine atom on the structural parameters of the coordination polyhedron becomes more evident with respect to bond angles. Thus, the angle between the two water molecules [78.26 (7)°, Hal = Br] is slightly reduced [79.9 (1)°, Hal = Cl]. This is also valid for the angle between the bromine atoms being *trans* to each other [170.32 (1)°, 1, 171.47 (3)°, 2]. In contrast, the angle [99.84 (1)°] between the bromine atoms *trans* to the water molecules is significantly enlarged in comparison to the corresponding angle between the chlorine atoms [93.54 (2)°] of 2.

Between the {SnBr_4_(H_2_O)_2_} octahedrons and the uncoordinated water molecules an extended array of hydrogen bonds of different strengths (Tab. 2) exist. The strongest ones are those of type O—H···O between the three different water molecules giving rise to a tube-like arrangement (Fig. 2) of these components. Within these *tubes*, the coordinated water molecule 1 acts as acceptor for one and donor for two hydrogen bonds. From the two other uncoordinated water molecules, molecule 2 serves as donor for one and acceptor for two hydrogen bonds, whereas molecule 3 is only acceptor for two hydrogen bonds. In addition, the hydrogen atoms of these two molecules not involved into O—H···O hydrogen bridges form weaker (longer) hydrogen bonds to bromine atoms. The shortest one of these O—H···Br hydrogen bridges interconnects neighboring *tubes* (Fig. 3) in the third dimension.

### Experimental

A suitable single-crystal was selected under a polarization microscope and mounted on a 50 µm MicroMesh MiTeGen Micromount ^TM^ using FROMBLIN Y perfluoropolyether (LVAC 16/6, Aldrich).

### Refinement

All H atoms of the water molecules were found in a difference Fourier synthesis. Their positions were first refined with respect to a O—H distance of 0.96 Å and an H—O—H angle of 104.95°. Thereafter their positions were constrained to ride on their parent oxygen atoms, with three common isotropic displacement parameter for the three different water molecules.

### Crystal data

**Table:**

[SnBr_4_(H_2_O)_2_]·3H_2_O	*F*(000) = 960
*M**_r_* = 528.41	*D*_x_ = 3.179 Mg m^−^^3^
Monoclinic, *C*2/*c*	Mo *K*α radiation, λ = 0.71073 Å
Hall symbol: -C 2yc	Cell parameters from 8700 reflections
*a* = 12.6484 (5) Å	θ = 2.6–30.0°
*b* = 10.1647 (4) Å	µ = 16.77 mm^−^^1^
*c* = 8.8683 (4) Å	*T* = 100 K
β = 104.462 (1)°	Block, colourless
*V* = 1104.04 (8) Å^3^	0.18 × 0.15 × 0.12 mm
*Z* = 4	

### Data collection

**Table:**

Bruker APEXII CCD diffractometer	1618 independent reflections
Radiation source: fine-focus sealed tube	1523 reflections with *I* > 2σ(*I*)
Graphite monochromator	*R*_int_ = 0.033
φ and ω scans	θ_max_ = 30.0°, θ_min_ = 3.2°
Absorption correction: multi-scan (*SADABS*; Bruker, 2009)	*h* = −17→16
*T*_min_ = 0.148, *T*_max_ = 0.234	*k* = −12→14
16999 measured reflections	*l* = −12→12

### Refinement

**Table:**

Refinement on *F*^2^	Secondary atom site location: difference Fourier map
Least-squares matrix: full	Hydrogen site location: inferred from neighbouring sites
*R*[*F*^2^ > 2σ(*F*^2^)] = 0.013	H-atom parameters constrained
*wR*(*F*^2^) = 0.028	*w* = 1/[σ^2^(*F*_o_^2^) + (0.0089*P*)^2^ + 1.3814*P*] where *P* = (*F*_o_^2^ + 2*F*_c_^2^)/3
*S* = 1.10	(Δ/σ)_max_ = 0.001
1618 reflections	Δρ_max_ = 0.46 e Å^−^^3^
51 parameters	Δρ_min_ = −0.41 e Å^−^^3^
0 restraints	Extinction correction: *SHELXL97* (Sheldrick, 2008), Fc^*^=kFc[1+0.001xFc^2^λ^3^/sin(2θ)]^-1/4^
Primary atom site location: structure-invariant direct methods	Extinction coefficient: 0.00035 (4)

### Special details

**Table:**

Geometry. All e.s.d.'s (except the e.s.d. in the dihedral angle between two l.s. planes) are estimated using the full covariance matrix. The cell e.s.d.'s are taken into account individually in the estimation of e.s.d.'s in distances, angles and torsion angles; correlations between e.s.d.'s in cell parameters are only used when they are defined by crystal symmetry. An approximate (isotropic) treatment of cell e.s.d.'s is used for estimating e.s.d.'s involving l.s. planes.
Refinement. Refinement of *F*^2^ against ALL reflections. The weighted *R*-factor *wR* and goodness of fit *S* are based on *F*^2^, conventional *R*-factors *R* are based on *F*, with *F* set to zero for negative *F*^2^. The threshold expression of *F*^2^ > σ(*F*^2^) is used only for calculating *R*-factors(gt) *etc*. and is not relevant to the choice of reflections for refinement. *R*-factors based on *F*^2^ are statistically about twice as large as those based on *F*, and *R*- factors based on ALL data will be even larger.

### Fractional atomic coordinates and isotropic or equivalent isotropic displacement parameters (Å^2^)

**Table:**

	*x*	*y*	*z*	*U*_iso_*/*U*_eq_	
Sn1	0.0000	0.259481 (16)	0.2500	0.00769 (5)	
Br1	0.143871 (16)	0.280603 (17)	0.09578 (2)	0.01115 (5)	
Br2	0.117864 (16)	0.098459 (17)	0.43896 (2)	0.01257 (5)	
O1	0.07836 (11)	0.42263 (12)	0.38481 (14)	0.0123 (3)	
H11	0.1025	0.4275	0.4963	0.048 (6)*	
H12	0.1063	0.5008	0.3480	0.048 (6)*	
O2	0.13528 (11)	0.39033 (13)	0.70061 (15)	0.0134 (3)	
H21	0.0954	0.3121	0.7118	0.038 (5)*	
H22	0.2103	0.3632	0.7231	0.038 (5)*	
O3	1.0000	0.19063 (19)	0.7500	0.0170 (4)	
H3	0.9756	0.1311	0.6643	0.056 (10)*	

### Atomic displacement parameters (Å^2^)

**Table:**

	*U*^11^	*U*^22^	*U*^33^	*U*^12^	*U*^13^	*U*^23^
Sn1	0.00819 (9)	0.00818 (8)	0.00695 (8)	0.000	0.00236 (6)	0.000
Br1	0.01097 (10)	0.01404 (9)	0.00975 (8)	0.00156 (7)	0.00504 (7)	0.00131 (6)
Br2	0.01312 (10)	0.01227 (9)	0.01191 (9)	0.00208 (6)	0.00235 (7)	0.00356 (6)
O1	0.0151 (7)	0.0124 (6)	0.0087 (6)	−0.0042 (5)	0.0017 (5)	−0.0013 (5)
O2	0.0131 (7)	0.0143 (6)	0.0126 (6)	0.0033 (5)	0.0027 (5)	0.0009 (5)
O3	0.0250 (12)	0.0150 (9)	0.0102 (9)	0.000	0.0026 (8)	0.000

### Geometric parameters (Å, º)

**Table:**

Sn1—O1^i^	2.1378 (12)	O1—H11	0.9600
Sn1—O1	2.1378 (12)	O1—H12	0.9600
Sn1—Br2^i^	2.5420 (2)	O2—H21	0.9600
Sn1—Br2	2.5420 (2)	O2—H22	0.9600
Sn1—Br1	2.5439 (2)	O3—H3	0.9602
Sn1—Br1^i^	2.5439 (2)		
			
O1^i^—Sn1—O1	78.26 (7)	O1^i^—Sn1—Br1^i^	86.63 (4)
O1^i^—Sn1—Br2^i^	90.98 (4)	O1—Sn1—Br1^i^	85.86 (4)
O1—Sn1—Br2^i^	169.06 (4)	Br2^i^—Sn1—Br1^i^	91.620 (7)
O1^i^—Sn1—Br2	169.06 (4)	Br2—Sn1—Br1^i^	94.613 (7)
O1—Sn1—Br2	90.98 (4)	Br1—Sn1—Br1^i^	170.317 (10)
Br2^i^—Sn1—Br2	99.837 (10)	Sn1—O1—H11	126.8
O1^i^—Sn1—Br1	85.86 (4)	Sn1—O1—H12	127.6
O1—Sn1—Br1	86.63 (4)	H11—O1—H12	105.0
Br2^i^—Sn1—Br1	94.612 (7)	H21—O2—H22	105.0
Br2—Sn1—Br1	91.621 (7)		

Symmetry code: (i) −*x*, *y*, −*z*+1/2.

### Hydrogen-bond geometry (Å, º)

**Table:**

*D*—H···*A*	*D*—H	H···*A*	*D*···*A*	*D*—H···*A*
O3—H3···Br2^ii^	0.96	2.67	3.5239 (16)	148
O2—H22···Br2^iii^	0.96	2.92	3.6365 (14)	133
O2—H22···Br1^iii^	0.96	2.58	3.4004 (13)	144
O2—H21···O3^iv^	0.96	1.82	2.7595 (19)	167
O1—H12···O2^v^	0.96	1.82	2.7182 (18)	155
O1—H11···O2	0.96	1.80	2.7318 (18)	164

Symmetry codes: (ii) −*x*+1, −*y*, −*z*+1; (iii) −*x*+1/2, −*y*+1/2, −*z*+1; (iv) *x*−1, *y*, *z*; (v) *x*, −*y*+1, *z*−1/2.
